# Molecular characterization of sheeppox virus from outbreaks in Karnataka, India

**DOI:** 10.14202/vetworld.2020.386-391

**Published:** 2020-02-28

**Authors:** K. Sumana, Yogisharadhya Revanaiah, R. Apsana, Parimal Roy, G. B. Manjunatha Reddy

**Affiliations:** 1 Indian Council of Agricultural Research Institute-National Institute of Veterinary Epidemiology and Disease Informatics, Yelahanka, Karnataka, India; 2 Department of Microbiology and Biotechnology, Jain University, Bengaluru, Karnataka, India

**Keywords:** goatpox, P32, polymerase chain reaction, phylogenetic analysis, sheeppox

## Abstract

**Aim:**

This study aimed to characterize sheeppox virus (SPPV) using the P32 gene of the *Capripoxvirus* (CaPVs).

**Materials and Methods:**

Clinical samples of skin, scabs, and nasal swab from suspected outbreaks Horalagallu (n=13) and Gerahalli (n=11) at Ramanagara district in Karnataka were collected. All the samples were initially subjected to genus-specific diagnostic polymerase chain reaction (PCR). The pooled clinical samples from each outbreak were also subjected to virus isolation. The isolates were confirmed by CaPVs genotyping PCR targeting the full-length P32 gene, followed by sequencing and phylogenetic analysis.

**Results:**

The clinical signs and lesions varied from mild to severe degree with no specificity between age and sex. Specific cytopathic changes in cell morphology were observed in infected Vero cells from both outbreaks, which were confirmed by PCR. The complete P32 gene from two outbreaks was successfully amplified with the expected amplicon size of 1006bp. The sequencing and phylogenetic analysis revealed that both the outbreaks were due to SPPV and shared high similarity with published SPPVs from Karnataka and other parts of India.

**Conclusion:**

The current study showed that complete P32 gene-based genotypic PCR assay can be used for genetic characterization and molecular epidemiology of both sheeppox and goatpox diseases and also to differentiate the causative agents. The sequence analysis revealed 100% similarity among the two outbreak isolates suggesting the same strain of the virus and common source of infection for the outbreaks.

## Introduction

Sheep and goats contribute greatly to the income of marginal and landless farmers of the farming community. India has the second largest population of goats (148.88 million) and fourth largest population of sheep (74.26 million) increased by 14.1% and 10.1%, respectively, over the previous census [1]. There are many diseases which affect sheep and goats, among them sheeppox and goatpox are one of the major diseases and cause severe economic loss in terms of damage to skin/hide and wool, reduced milk yield, and mortality [2,3]. The disease in sheep and goat is caused by sheeppox virus (SPPV) and goatpox virus (GTPV), respectively. The viruses belong to the genus *Capripoxvirus* (CaPVs) of the family *Poxviridae* [4,5]. Sheep and goatpox disease is enzootic in Northern and Central Africa and in Asia including the Indian subcontinent [6,7]. The mortality rate in a susceptible population of sheep and goatpox may reach up to 50% in adults and 100% in the young stock and morbidity rate can reach up to 100% [8]. Sheeppox and goatpox outbreaks are being increasingly reported from different parts of India [9].

The virions of Capripox are ovoid in shape with an average size of 294 nm × 273 nm. Their genome is linear, double-stranded DNA of ~150 kb, contains 156 putative genes with high adenine and thymine (AT) content of 73-75% and shares 96% nucleotide (nt) identity. Among the 156 open reading frames (ORFs), the conserved essential genes of replication, structure, and assembly are located in the middle region (ORFs 024 to 123) and terminal variable region (ORFs 01 to 023 and 124 to 156) responsible for virulence and host range functions [10]. All the CaPVs having a structural protein called P32, contain a major immunogenic determinant [11]. CaPVs are currently classified within the genus based on the animal species from which the viruses are isolated. However, some SPPV and GTPV isolates found to be caused infection in both sheep and goats [12,13]. As both SPPV and GTPV are antigenically closely related and show similar clinical signs hence, they cannot be differentiate based on serological methods [14]. Recent molecular studies have shown that CaPVs are phylogenetically and genetically distinct based on individual and whole-genome sequencing [15-17].

The present study was undertaken to differentiate and characterize the SPPV isolates from field outbreaks.

## Materials and Methods

### Ethical approval

Ethical approval was not necessary for this study. However, samples were collected as per the standard sample collection procedure without any unnecessary harm or stress to the animals.

### Outbreak history and sample collection

The disease outbreaks were reported during March 2016 from two mixed unvaccinated flocks consisting of Horalagallu and Gerahalli villages of Ramanagara district, Karnataka. The flocks consisted of local breeds of sheep and goats of young and adult age groups of both the sex. The disease was observed only in sheep, but in goats, there were no signs of disease. The clinical samples of nasal swab, skin lesion, and scabs were collected and were transported to the laboratory.

### Virus isolation

The skin scabs and nasal swabs were triturated with 1 ml of sterile phosphate-buffered saline, followed by 3 times freeze-thawing. The triturate was incubated with an antibiotic and antimycotic solution for 1 h at 37°C, followed by centrifugation at 2000 rpm for 10 min. The supernatant was filtered with 0.45-micron syringe filter. Approximately 500 μl of inoculum was added to a confluent monolayer of Vero cells by 1 h adsorption method at 37°C to allow virus attachment; the inoculum was decanted and fresh maintenance medium (Dulbecco’s Modified Eagle’s Medium) was added. The cells were incubated at 37°C with 5% CO_2_ and examined periodically. Infected flasks were given six blind passages at weekly intervals until cytopathic effect (CPE), which could be observed in seventh passage. The flasks showing CPE such as rounding, clumping, and detachment were freeze-thawed 3 times and were further processed for virus confirmation.

### DNA extraction and amplification

The DNA was isolated from infected cells from both the outbreaks (Horalagallu-Nasal swab, Gerahalli-Skin scab) by DNeasy Blood and Tissue Kit (Qiagen, Germany), as per the manufacturing protocol. The purity and quantity of extracted DNA was determined by spectrophotometer. DNA was initially subjected to a diagnostic polymerase chain reaction (PCR) for the identification of genus-specific CaPVs by amplification of partial P32 gene for virus confirmation. Later same DNA samples were subjected to genotypic PCR by targeting full-length P32 gene (Table-1) [18,19]. The PCR was carried out 25 µl reaction volume containing 12.5 µl of Dream Taq Green PCR Master Mix (Thermo Fisher Scientific, USA), 10 pM of each forward and reverse primer, 7.5 µl of nuclease-free water, and 3 µl of extracted DNA with following conditions of initial denaturation at 94°C for 5 min, then 35 cycles of 94°C for 1 min, 57°C for 1 min, and 72°C for 1 min followed by final extension at 72°C for 10 min in thermocycler (S1000 thermal cycler, Bio-Rad). The specific amplification was confirmed by agarose gel electrophoresis (1% agar) using a gel documentation system (Syngene, Biodigital Pvt Ltd).

**Table-1:** List of primers used for partial and complete gene amplification of P32.

Primer name	Sequence (5’- 3’)	Length	Tm (°C)	Product length (bp)	References
SGPP32FP	ACACAGGGGGATATGATTTTACC	23	52	237	[18]
SGPP32RP	ATACCGTTTTTCATTTCGTTAGC	23			
B7-Forward	AACACTCTCATTGGTGTTCGG	21	57	1006	[19]
A95-Reverse	CACATGGCAGATATCCCATTA	21			

### Sequencing and phylogenetic analysis

The gel slices containing PCR amplicons were purified using the GeneJET Gel Extraction Kit (Thermo Fisher Scientific, USA). The eluted products were cloned into pGEM-T vector cloning kit (Promega) and transformed into the top ten *Escherichia coli* cells. Blue-white screening was observed by adding X-gal (100 mg/µl) and Isopropyl β- d-1-thiogalactopyranoside (50 mg/µl) on Luria Broth plate. Recombinant clones were confirmed by colony PCR using gene-specific primers. The plasmid DNA was extracted using the plasmid DNA extraction kit (Thermo Fisher Scientific, USA) and sequenced (Eurofins, Bengaluru). The forward and reverse sequences were edited and submitted to GenBank. Sequences obtained from the current study and other GenBank sequences were aligned by ClustalW using the neighbor-joining method [20] and the phylogenetic tree was constructed using MEGA 10.0.5 [21].

## Results and Discussion

Sheeppox and goatpox diseases of the Capripox genus are enzootic in India and are accountable for high economic losses due to high morbidity and mortality. The sheeppox disease outbreaks were reported in two separate mixed (sheep and goats) flocks from two different villages under Kanakapura Tehsil, Ramanagara district of Karnataka were included in the study. The morbidity and mortality rate recorded were higher in Gerahalli with 75% (60/80) and 55% (33/60) compared to Horalagallu village with 66.66% (40/60) and 52.5% (21/40), respectively (Table-2). The variable morbidity and mortality rates are mainly attributed to host species and virus isolate, pox disease [8]. On personal interaction with the farming community, it was learned that the pox outbreaks are rarely encountered. However, the outbreaks coincide with less rainfall and fodder shortage during which there is very probability of migration of flocks. Due to the less prevalence of pox disease in the past, the farmers did not practice regular vaccination. However, in spite of community grazing and water facility, the other flocks in the villages did not report the disease due to the practice of regular vaccination. During the present outbreak, only sheep were affected even though in both villages, the herds affected had goats along with sheep. Off late, the SPPVs and GTPVs infections are being reported with host specificity [22], whereas some strains equally infect both the species, but the disease severity and pattern caused by the same isolate may vary between sheep and goats [23,24].

**Table-2:** Animal population and disease prevalence of two outbreak flocks.

Animals	Sheep							Goat details of outbreak	

	Details of outbreak			Number of clinical samples collected		Number of clinical samples used			

	Flock size	Morbidity	Mortality	Nasal swab	Skin scab	Nasal swab	Skin scab	Flock size	Morbidity
Flock: 1-horalagallu	60	40	21	5	8	2	2	20	0
Flock: 2-gerahalli	80	60	33	4	7	2	2	5	0

**Figure-1:**
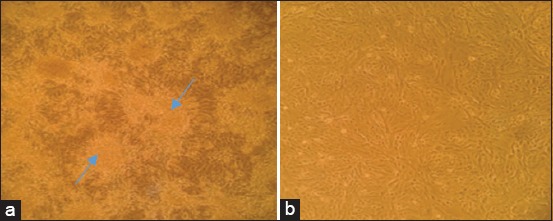
Virus isolation: Vero cells showing characteristic cytopathic effects such as rounding, clumping, and finally detachment of the cells were observed at seventh passage (a) compared to healthy (b).

**Figure-2:**
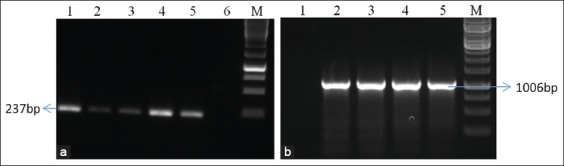
Agarose electrophoresis: The clinical samples were subjected to a partial P32 gene showing specific amplification at 237bp band. M-1kb marker, L1-L4 – outbreak samples, L5-positive control, and L6 Negative control (a). Isolated samples subjected to full-length P32 gene with specific amplification at 1006bp. M-1kb marker, L1 - Negative control, L2, and L3 - outbreak samples, L4 and L5 - positive control (b).

In both, the outbreaks, clinical signs such as fever, dullness, anorexia, vesicles, loss of wool, alofacia, mucopurulent nasal/oral discharge, edema of eyelids, conjunctivitis, nodules, and pox lesions were observed in the hairless region of the body including muzzle, udder, teats, ventral part of the tail, lips, and on ears were recorded with variable degree similar to earlier reports [23,25]. Clinical samples (Nasal;4/6, Scab;25/25) were found positive for the partial P32 gene by preliminary screening by PCR [18]. The polled samples from both nasal and scabs showed characteristic cytopathic changes such as rounding of cells, clumping, and finally detachment of cells from the surface in Vero cells (Figure-1). The CPE was observed after sixth blind passage, i.e, seventh passage in Vero cells. The growth of the virus was first confirmed by genus-specific PCR with a specific amplicon of size 237bp. Further to know species of virus isolates, two isolates (one for each outbreak) were subjected for complete ORF of P32 gene PCR (Figure-2), followed by sequencing. The species of the virus was confirmed as SPPV by sequence analysis.

**Figure-3:**
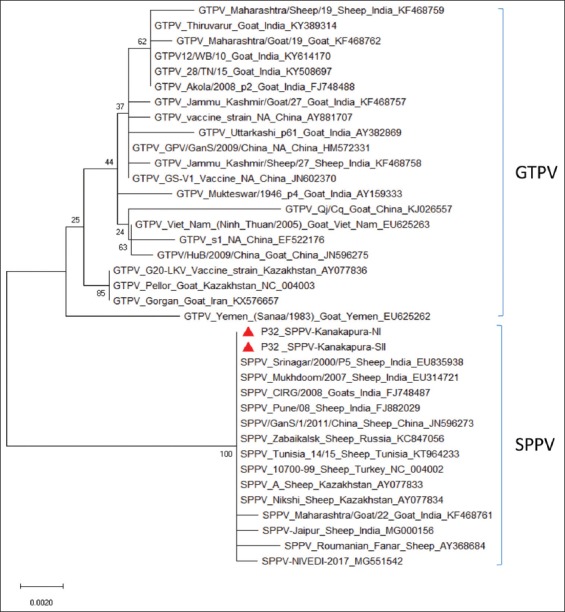
Phylogenetic tree: Phylogenetic tree based full-length P32 gene nucleotide sequence of sheeppox virus (SPPV) isolate from outbreak was carried out by the neighbor–joining program using MEGA version 10.0.5 (bootstrap 1000). Current outbreak sequences of SPPV were indicted by the red color triangle shape.

**Figure-4:**
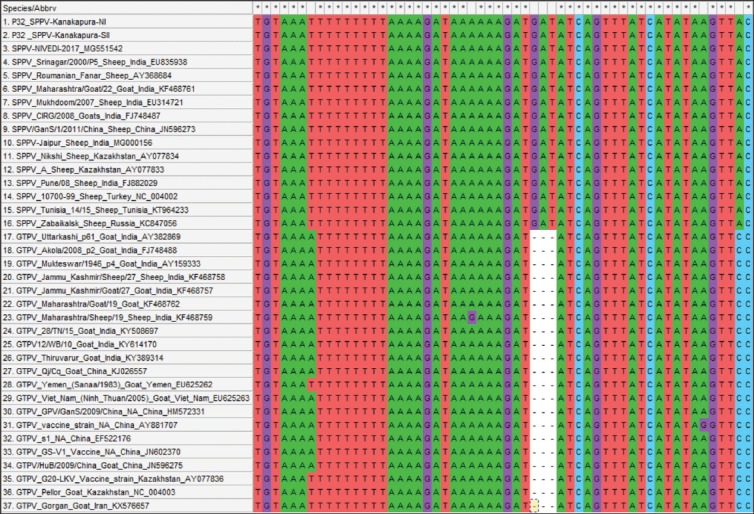
Nucleotide (nt) variation between sheeppox virus (SPPV) and goatpox virus (GTPV) viruses: Presence of three nt at 163:G, 164:A, and 165:T positions in SPPV, which are deleted in GTPV gene encoding for major envelope protein (P32).

The complete P32 gene sequences obtained were annotated at 972 nts using online tool BLAST-n and GenBank accessions were obtained (MK607146 and MN639777). The sequence analysis revealed both the isolates belong to SPPV with the presence of SPPV specific nts and amino acid (aa) as compared to GTPV. The phylogenetic analysis showed that both the sequences from present outbreaks were grouped into SPPVs clade compared to other poxviruses. There were two separate clades of CaPVs with SPPV isolates forming separate groups from that of GTPV isolates (Figure-3). The P32 sequence analysis of both SPPVs and GTPVs revealed the presence of three specific nts for SPPV, which is deleted in the case of GTPVs (Figure-4), confirming the present outbreaks were due to SPPV. The multiple alignments revealed that isolates of both outbreaks shared 100% with each other at nt and aa levels indicating same strain of SPPV causing the outbreaks in both the villages. This was also supported by the observation of farmers that the flocks share common grazing land and water during summer session along with migratory flocks. The isolates also a very high similarity with previously isolated SPPVs at the nt and aa from Karnataka, followed by India. On the global level, the present isolates were closely related to SPPV isolates of China as reported earlier [18,26].

## Conclusion

The present outbreaks were attributed to the same strain of SPPV based on clinical, virus isolation, host specificity, and sequence analysis. The present kind of outbreaks with high morbidity and mortality warrants the need for prevention and control of sheep and goatpox disease through regular vaccination and monitoring the host specificity of CaPVs for the improvement of specific homologous vaccines in future for preventing the economic loss to the farming community.

## Authors’ Contributions

The present study is the part of KS’s Ph.D., dissertation work. KS, GBMR, and YR visited the field, collected the samples, KS and RA carried out the laboratory experiment, SK drafted the manuscript. GBMR, PR, and YR designed the work, provided guidance with overall monitoring, analyzed the data and edited the manuscript. The final manuscript was drafted, read, and approved by all the authors.
